# Dynamic changes of serum taurine and the association with gestational diabetes mellitus: A nested case-control study

**DOI:** 10.3389/fendo.2023.1116044

**Published:** 2023-03-23

**Authors:** Jia Wang, Yuanyuan Wang, Wei Zheng, Xianxian Yuan, Cheng Liu, Ya Zhang, Wei Song, Xiaoxin Wang, Shengnan Liang, Xu Ma, Guanghui Li

**Affiliations:** ^1^ Division of Endocrinology and Metabolism, Department of Obstetrics, Beijing Obstetrics and Gynecology Hospital, Capital Medical University, Beijing Maternal and Child Health Care Hospital, Beijing, China; ^2^ National Research Institute for Family Planning, Beijing, China; ^3^ National Human Genetic Resources Center, Beijing, China

**Keywords:** biomarker, gestational diabetes mellitus, taurine, taurine transporter, dynamic change

## Abstract

**Objective:**

There is a lack of risk factors that can effectively identify gestational diabetes mellitus (GDM) in early pregnancy. It is unclear whether serum taurine in the first trimester and dynamic changes have different characteristics in GDM women. Whether these features are associated with the occurrence of GDM has not yet been elucidated. The main objective of this study was to observe the dynamic changes of serum taurine during pregnancy and investigate the relationship between serum taurine levels and GDM in the first and second trimesters.

**Methods:**

This was a nested case-control study in 47 women with GDM and 47 age-matched normoglycemic women. We examined serum taurine at 8-12 weeks’ gestation and 24-28 weeks’ gestation. The serum taurine of the two groups was compared. Multivariable logistic regression analysis was performed to investigate how serum taurine was associated with GDM.

**Results:**

The serum taurine concentration of GDM women was significantly lower than that of normoglycemic women in the first trimester(2.29 vs 3.94 μmol/L, P<0.001). As the pregnancy progressed, serum taurine concentration in normoglycaemic women decreased significantly(3.94 vs 2.47 μmol/L, P<0.001), but not in the GDM group(2.29 vs 2.37 μmol/L, P=0.249), resulting in the disappearance of differences between the two groups(2.47 vs 2.37 μmol/L, P=0.160). After adjustment for pre-pregnancy body mass index(BMI), fasting plasma glucose(FPG), and lipid profiles in the first trimester, the serum taurine concentration in the first trimester was negatively correlated with the risk of GDM(OR=0.017, 95% CI=0.003-0.107, P<0.001). Furthermore, dynamic change of serum taurine showed a significantly positive correlation with the risk of GDM(OR=9.909, 95% CI=3.556-27.610, P<0.001).

**Conclusion:**

Low serum taurine concentration in the first trimester was significantly associated with the development of GDM. As the pregnancy progressed, the association between serum taurine and GDM disappeared in the second trimester, which might be related to the inhibition of taurine transporter(TauT) activity by high glucose.

## Introduction

Gestational diabetes mellitus (GDM) is the most common metabolic disease in pregnancy, with an incidence of 9%-25% globally according to the International Diabetes Federation (IDF) (1). Women with GDM are at an increased risk of gestational hypertension, pre-eclampsia, and cesarean section, as well as long-term risk of type 2 diabetes (T2DM) and cardiovascular disease (2). Maternal hyperglycemia will increase the risk of large for gestational age(LGA), shoulder dystocia or birth injury, and neonatal hypoglycemia (3). The offspring of GDM women are at increased long-term risk of obesity, abnormal glucose metabolism, and cardiovascular disease (4). With the continuous progress in knowledge of GDM, the oral glucose tolerance test (OGTT) at 24-28 gestational weeks was the diagnostic criteria for GDM (2). Recent studies evaluating maternal glycemia in relation to fetal growth trajectory have confirmed the early impact of maternal glycemia on fetal overgrowth and obesity prior to the diagnosis of standard GDM (5, 6). Lifestyle interventions such as dietary counseling or physical activity in the first trimester were demonstrated to effectively reduce the incidence of GDM and its associated adverse pregnancy outcomes (7, 8). As a result, it is of great clinical value to identify risk factors for GDM, especially in the first trimester.

Taurine which is the most abundant free amino acid in the human body and the key component of bile acid has many biological effects such as antioxidant, anti-inflammatory, improvement of insulin resistance(IR), neuroprotection, and anti-neurotoxicity (9, 10). Taurine can be made endogenous from cysteine or methionine, provided extrinsic from the diet, or affected by gut microbiota (11, 12). There was a significant negative correlation between taurine and non-gestational blood glucose, and taurine supplementation was effective in improving diabetes and other chronic metabolic diseases and preventing related complications (10). A recent study suggested a lower plasma taurine level in the first trimester seemed to be a fair marker of inadequate insulin secretion and to be more closely associated with a higher risk of GDM development in multiparas (13). However, the dynamic changes in serum taurine from the first to second trimester were unknown.

The main objective of this study was to observe the dynamic changes of serum taurine during pregnancy and investigate the relationship between serum taurine levels and GDM in the first and second trimesters.

## Materials and methods

### Patient cohorts

**Figure 1:**
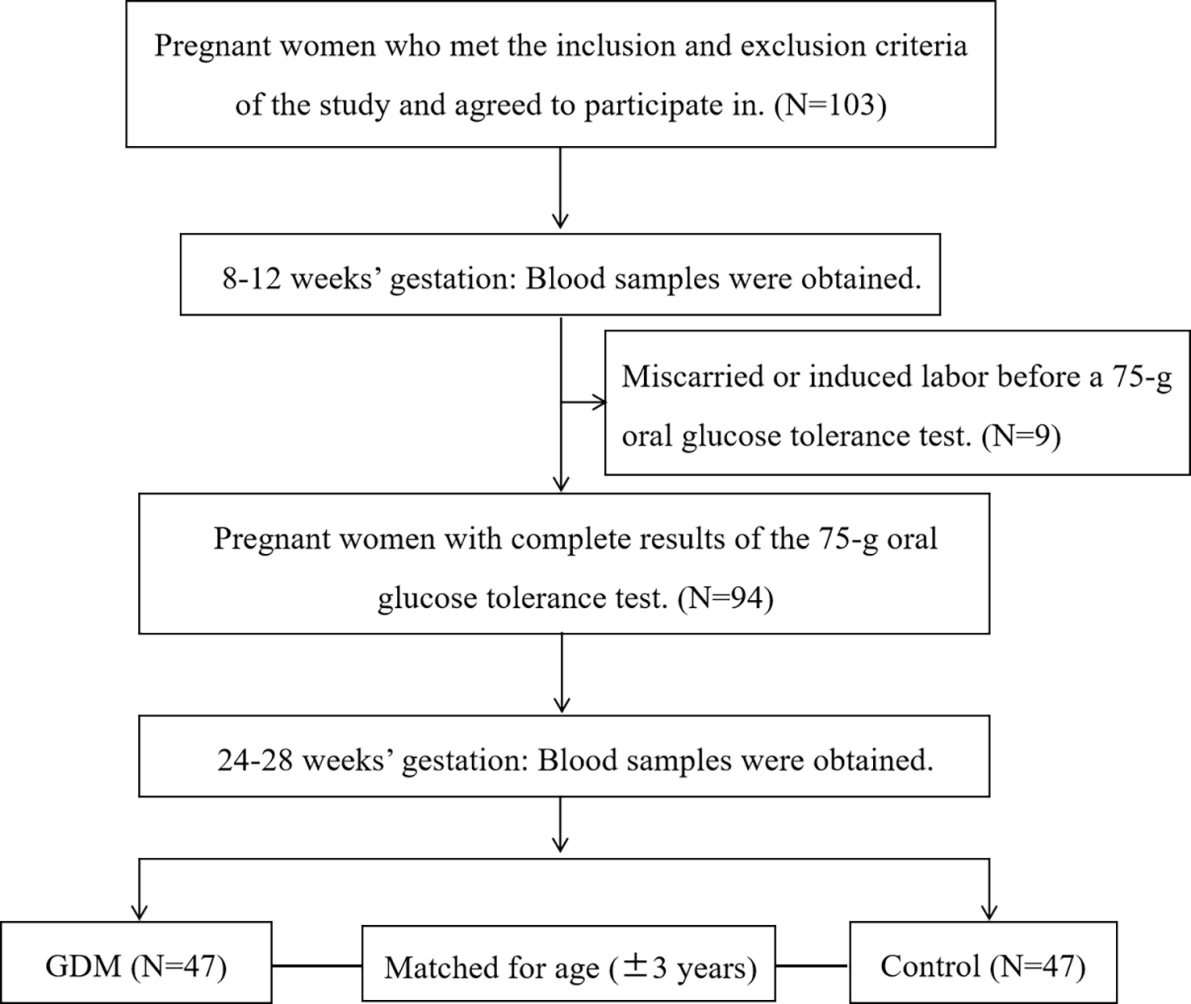
Flowchart of the included participants in this study.

The participants in this nested case-control study were from a prospective cohort study in the Beijing Obstetrics and Gynecology Hospital, Capital Medical University. All pregnant women who intended to give birth in this hospital were enrolled in the cohort study at 8-12 gestational weeks and followed up until delivery. To evaluate the relationship between serum taurine and GDM, we selected eligible subjects from the recruited pregnant women above. Singleton pregnant women aged 18 to 44 years were recruited and only participants with complete clinical information were included in the analysis. Women with hypertension, diabetes, hyperlipidemia, liver or kidney dysfunction, and infectious diseases (hepatitis, pulmonary tuberculosis, etc.) before pregnancy were excluded. A 75-g OGTT was carried out at 24-28 gestational weeks. The diagnosis of GDM was made when any one of the following values was met or exceeded in the 75-g OGTT: 0 h (fasting), 5.1 mmol/L; 1 h, 10.0 mmol/L; and 2 h, 8.5 mmol/L according to ADA criteria (14). Normoglycaemic women were matched for age ( ± 3 years) to each case of GDM women in the same cohort (**Figure 1**).

### Clinical measurements and covariates

Anthropometric measurements of participants were completed by trained medical staff at recruitment using a standardized protocol. Clinical data were collected by medical record review. Pre-pregnancy body weight was self-reported. A family history of diabetes was defined as a first-degree relative with T2DM. The fasting plasma glucose(FPG) and lipid profiles, including cholesterol (TC), triglyceride (TG), high-density lipoprotein (HDL), and low-density lipoprotein (LDL), were determined as described in a previous study (15).

### Taurine examination

Blood samples were collected from participants following an overnight fast at 8-12 weeks and 24-28 weeks, and serum specimens were isolated and stored at -80°C for further examination. The serum taurine levels were examined by liquid chromatography coupled to tandem mass spectrometry (LC-MS/MS, Thermo Scientific, USA). First, 100 μL of human serum was briefly added to a 0.5 mL glass centrifuge tube. After centrifugation at 14000 r/min for 5 min, the serum sample was dried under nitrogen at 50°C. Then, 60 μL of N-butyl alcohol and 12 mol/L HCI (95:5, v/v) were added and vortexed for 30 seconds in a seal. After incubation at 65°C for 15 min for derivatization, the derivatized solution was centrifuged, and dried under nitrogen at 50°C again. The residue was reconstituted by adding 100 μL of acetonitrile and water (4:1, v/v), vortexed for 30 seconds, centrifuged at 14000 r/min for 5 min, and injected at 20 μL for LC-MS/MS analysis.

### Sample size calculation

The sample size was calculated using the mean and standard deviation of serum taurine in two groups. The test level (α) was 0.05, and the power (1-β) was 0.8. Serum taurine concentrations are 0.6 ± 0.1 mmol/L in diabetic patients and 0.8 ± 0.2 mmol/L in healthy adults (16). The minimum sample size was 48, and the sample size of this study was 94, which was sufficient according to the sample size calculation.

### Statistics

Data were analyzed using the SPSS 26.0 software. Data with normal distributions were shown as the mean ± standard deviation, and nonnormal distributed data were shown as the median (interquartile range), respectively. T-tests and Wilcoxon tests were used to analyze the differences in continuous variables between the GDM group and the control group. Serum taurine concentrations were also compared by t-test. Categorical variables, including serum taurine levels (categorized into quartiles), were evaluated using the Cochran-Armitage. As pre-pregnancy body mass index(BMI) remained higher in the GDM group, we adjusted for pre-pregnancy BMI when comparing serum taurine levels in the two groups. Binary logistic regression for the association between GDM and serum taurine was carried out with adjustment for potentially confounding variables. The results are represented by the odds ratio (OR) and 95% confidence interval (CI). The differences were considered statistically significant when P<0.05.

## Results

### Clinical and laboratory characteristics

The study included 47 GDM women and 47 normoglycemic women. There was no history of GDM, macrosomia, or low birth weight delivery in both two groups. Pre-pregnancy BMI was significantly higher in the GDM women(22.32 vs 20.67, p=0.001), and other clinical indicators were similar, including gravidity, primipara, and history of polycystic ovary syndrome. However, FPG and lipid profiles including TC, TG, and LDL, were significantly higher among GDM women in the first trimester(FPG: 4.86 vs 4.64mmol, P=0.017; TC: 4.46 vs 4.12, P=0.021; TG: 1.26 vs 1.02, P=0.023; LDL: 2.33 vs 2.08, P=0.025)(**Table 1**). At OGTT, the blood glucose value of the GDM group was significantly higher, but there was no difference in lipid profiles between the two groups in the second trimester (**Table S1** in the supplemental material).

**Table 1:** Baseline characteristics and glycolipids metabolism in the first trimester between two groups.

	GDM (n=47)	Control (n=47)	P-value
Age (year)	33.0 ± 3.61	32.1 ± 2.91	0.170
Gravidity (first pregnancy)	21(44.68%)	24(51.06%)	0.536
Primipara	30(63.83%)	32(68.09%)	0.663
Smoking	2(4.55%)	1(2.13%)	0.608
Alcohol consumption	5(11.36%)	4(8.51%)	0.734
History of adverse pregnancy outcomes	8(17.02%)	3(6.38%)	0.156
History of PCOS	3(6.38%)	0(0.00%)	0.242
Family history of hypertension	13(29.55%)	11(23.40%)	0.506
Family history of diabetes	11(23.40%)	6(12.77%)	0.180
Pre-pregnancy BMI (kg/m^2^)	22.32 ± 2.72	20.67 ± 2.03	0.001
FPG (mmol/L)	4.86 ± 0.49	4.64 ± 0.36	0.017
TC (mmol/L)	4.46 ± 0.74	4.12 ± 0.65	0.021
TG(mmol/L)	1.26(0.73)	1.02(0.42)	0.023
HDL(mmol/L)	1.48 ± 0.30	1.48 ± 0.29	0.922
LDL(mmol/L)	2.33 ± 0.61	2.08 ± 0.48	0.025

History of adverse pregnancy outcomes included spontaneous abortion, preterm, stillbirth, delivery of deformities, and early neonatal death. PCOS, polycystic ovary syndrome; FPG, fasting plasma glucose; TC, cholesterol; TG, triglycerides; HDL, high-density lipoprotein; LDL, low-density lipoprotein.

### Serum taurine levels between or within GDM and normoglycemic women

**Figure 2:**
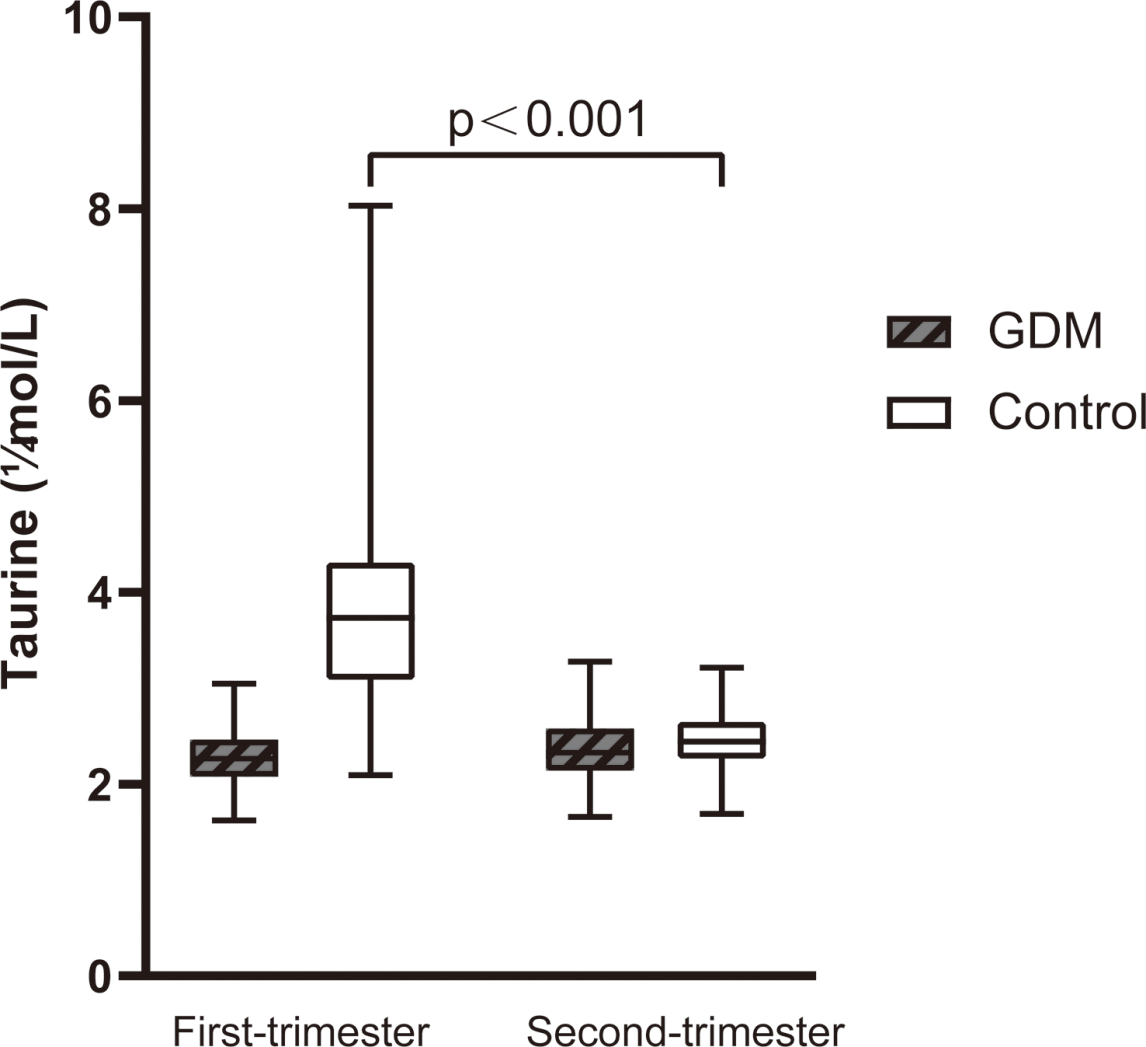
The dynamic changes of serum taurine between the first and second trimester of women with GDM and controls. Serum taurine concentration in control decreased significantly (P<0.001).

We compared serum taurine concentrations of GDM women and normoglycemic women at different stages of pregnancy, as well as the dynamic changes of serum taurine in the two groups(**Table 2**). The serum taurine concentration of GDM women was significantly lower than that of normoglycemic women in the first trimester(2.29 vs 3.94 μmol/L, P<0.001). When stratified by quartile, there were 23 controls and no GDM women with a taurine concentration less than 2.22 and there were 2 controls and 21 GDM women with a taurine concentration greater than 3.74(P<0.001). The serum taurine concentration was similar between the two groups in the second trimester(2.37 vs 2.47 μmol/L, P=0.147), and there was no significant difference in quartile stratification(P=0.064). With the progress of pregnancy, serum taurine concentration decreased significantly in the control group(3.94 vs 2.47 μmol/L, P<0.001), but not in the GDM group(2.29 vs 2.37 μmol/L, P=0.249) (**Figure 2**).

**Table 2:** Serum taurine concentration and quartile stratification comparison between two groups.

	GDM (n=47)	Control (n=47)	T/R	P-value
Taurine in the first trimester (μmol/L)	2.29 ± 0.31	3.94 ± 1.32	-8.327	<0.001
Quartile Stratification			-0.749	<0.001
<2.22	21(44.68%)	2(4.26%)		
2.22-2.67	21(44.68%)	4(8.51%)		
2.68-3.74	5(10.64%)	18(38.30)		
>3.74	0(0.00%)	23(48.94%)		
Taurine in the first trimester (μmol/L)	2.37 ± 0.33	2.47 ± 0.34	-1.462	0.147
Quartile Stratification			-0.192	0.064
<2.19	14(29.79%)	9(19.15%)		
2.19-2.39	14(29.79%)	11(23.40%)		
2.40-2.62	11(23.40%)	12(25.53%)		
>2.62	8(17.02%)	15(31.91%)		
△Taurine	0.08 ± 0.49	-1.47 ± 1.44	6.983	<0.001

△Taurine, changes in taurine from the first to second trimester.

### The association between serum taurine and GDM

Univariate logistic regression analysis showed that there was a significant negative correlation between serum taurine concentration in the first trimester and the risk of GDM(OR=0.013, 95% CI=0.002-0.082, P<0.001, **Table 3**). Furthermore, dynamic change of serum taurine showed a significantly positive correlation with GDM(OR=11.098, 95% CI=4.085-30.155, P<0.001, P<0.001, **Table 3**). Results did not change after adjustment for pre-pregnancy BMI, FPG, and lipid profiles in the first trimester(Taurine in the first trimester: OR=0.017, 95% CI=0.003-0.107, P<0.001; ΔTaurine: OR=9.909, 95% CI=3.556-27.610, P<0.001; **Table 3**). However, serum taurine concentration in the second trimester was not correlated with GDM in any case.

**Table 3:** The relationship between Taurine and GDM.

	OR(95%CI)	P-value	Adjusted OR (95% CI)	P-value
Taurine in the first trimester	0.013(0.002-0.082)	<0.001	0.017(0.003-0.107)	<0.001
Taurine in the second trimester	0.400(0.115-1.387)	0.149	0.248(0.056-1.089)	0.065
△Taurine	11.098(4.085-30.155)	<0.001	9.909(3.556-27.610)	<0.001

△Taurine, changes in taurine from the first to second trimester. OR odds ratio, CI confdence interval. Adjusted OR, adjusted for pre-pregnancy BMI, FPG, TC, TG, LDL.

## Discussion

Our study showed that serum taurine concentration in the first trimester was significantly lower in women who were later diagnosed with GDM. As the pregnancy progressed, serum taurine concentration in normoglycaemic women decreased significantly, resulting in the disappearance of differences between the two groups. Low serum taurine concentration in the the first trimester was significantly associated with the occurrence of GDM, and this correlation also no longer existed in the second trimester.

A significant negative association between taurine and T2DM has been demonstrated (16). Previous RCT studies have shown that taurine supplementation could effectively improve metabolic indicators of T2DM, including glycemic indexes, lipid profiles, and inflammatory biomarkers, and prevent related complications (17–19). The T2DM patients in these studies were all detected with improvement in clinical metabolic markers after supplementing with 3000mg/day of taurine for 8 weeks. In addition, animal experiments showed that taurine had a protective effect on liver damage in GDM offspring (20). A study conducted the dietary survey at 24-28 gestational weeks and found that taurine intakes were lower in GDM than non-GDM in normal-weight women (21). However, there were few studies establishing a link between serum taurine levels and the risk of GDM. A recent study suggested a lower plasma taurine level in the first trimester seemed to be a fair marker of inadequate insulin secretion and to be more closely associated with a higher risk of GDM development in multiparas (13). This was consistent with our findings regarding the relationship between low serum taurine concentration in the first trimester and GDM.

Our study further compared the serum taurine concentrations in the second trimester and analyzed its dynamic changes. We found that as the pregnancy progressed, serum taurine concentration decreased significantly in normoglycaemic women but not in GDM women, resulting in the disappearance of differences between the two groups. The taurine decline trend from the first to second trimester was significantly negatively associated with the occurrence of GDM. Taurine is an amino acid that links the mother with the offspring during pregnancy, and fetuses depend on the taurine supplied by mothers *via* the placenta (22). The concentration of taurine in the placental tissue is 100-150 times higher than that of the fetus and mother (23). The placental tissues concentrate taurine efficiently and transfer taurine to fetal circulation based on the taurine transporter(TauT) activity (22). Animal studies have demonstrated that taurine concentration correlated with the peak of neurogenesis (24), which explained the decrease in serum taurine concentration in normoglycaemic women as the pregnancy progressed. However, high glucose levels could acutely inhibit taurine’s transport by TauT (25), which might be the reason why there was no difference in serum taurine concentration between the first and second trimester of GDM women in our study. The offspring of GDM have a long-term risk of neurodevelopmental disorder (26), and the role of taurine transport inhibition is worth further study.

The beneficial effects of taurine on T2DM and its related complications have been widely reviewed in human clinical practice (27). Taurine played a hypoglycemic role by improving insulin sensitivity, stimulating insulin secretion, and reducing inflammation and oxidative stress (27). Previous studies have reported the role of taurine in maintaining glucose homeostasis involving several possible mechanisms, such as modulating several pancreatic cells (28) and inhibiting inflammatory factor and nuclear factor kappa-B(NF-κB) activity to reduce inflammatory-mediated destruction of pancreatic β cells (29). It is not clear whether the pathogenesis of GDM induced by taurine deficiency in the first trimester is identical to that in non-pregnant women. In our former study, we reported gut microbiota changes in the first trimester were potentially associated with the development of GDM (30). The gut microbiota could trigger inflammatory processes by increasing gut permeability by exposing tight gap junction proteins to bacterial lipopolysaccharides (31, 32). Taurine is a microbiota-related metabolite derived from bile acids by certain microorganisms (33), and animal studies have shown that taurine has a protective effect on intestinal barrier function (34). Taurine deficiency might play a critical role in the pathogenesis of GDM, resulting in the loss of intestinal barrier protection and chronic inflammation. Although a direct causal relationship between taurine and its pathological state has not been established, it might be a potential marker for GDM. We hope to develop a sensitive and reliable GDM prediction model with serum taurine in the first trimester to help identify high-risk individuals at an early stage. In addition, the clinical intervention can be stratified according to the high-risk degree to avoid the waste of medical resources.

## Strengths and limitations

This was the first study to compare the dynamic changes of serum taurine concentrations from the first to second trimester. Our results demonstrated that small molecule metabolites varied during pregnancy and should be combined with dynamic changes to analyze their relationship with disease. Unfortunately, we were unable to collect umbilical cord blood to test their serum taurine levels to verify the relationship between taurine transport and the dynamic change of serum taurine concentration during pregnancy. In the future, the serum taurine levels of mothers and newborns could be detected simultaneously to reveal this correlation and its role in offspring nervous system development. In addition, this study was a single-center study, limited by the sample size and limited geographical area.

## Conclusion

Our study revealed that GDM women had a reduced serum taurine level in the first trimester. Elevated serum taurine concentration from the first to second trimester was significantly associated with the development of GDM. The relationship between taurine deficiency and GDM may be related to increased intestinal permeability and systemic inflammation, and the specific mechanism needs to be further explored.

## Data availability statement

The raw data supporting the conclusions of this article will be made available by the authors, without undue reservation.

## Ethics statement

The studies involving human participants were reviewed and approved by the Ethics Committee of Beijing Obstetrics and Gynecology Hospital. The patients/participants provided their written informed consent to participate in this study.

## Author contributions

All the authors contributed significantly to the manuscript. GL and XM contributed to the study design and interpretation of the data. JW and YW contributed to the drafting and revision of the manuscript. JW and WZ coordinated and executed the statistical analysis. CL, XY, and YZ contributed to the collection of data. WS, XW, and SL contributed to the enrollment and follow-up in clinic. All authors reviewed and approved the final submitted version.
