# Supplementary material for: Dynamic changes of serum taurine and the association with gestational diabetes mellitus: A nested case-control study
Source: Front Endocrinol (Lausanne). 2023 Mar 23;14:1116044. doi: 10.3389/fendo.2023.1116044 (PMC10076711; doi:10.3389/fendo.2023.1116044)
Supplement: Supplementary file 1 [file Table_1.docx]

Supplementary Material

Dynamic Changes of Serum Taurine and the Association with Gestational Diabetes Mellitus: A Nested Case-Control Study

Jia Wang1†, Yuanyuan Wang2,3†, Wei Zheng1, Xianxian Yuan1, Cheng Liu1, Ya Zhang2,3, Wei Song1, Xiaoxin Wang1, Shengnan Liang1, Xu Ma2,3* and Guanghui Li1*

^*^Corresponding Author: These authors contributed equally to this work share corresponding authorship.

Guanghui Li, Division of Endocrinology and Metabolism, Department of Obstetrics, Beijing Obstetrics and Gynecology Hospital, Capital Medical University. Beijing Maternal and Child Health Care Hospital, Beijing, China. No. 251, Yaojiayuan Road, Chaoyang District, Beijing, China, 100026. [liguanghui@ccmu.edu.cn](mailto:liguanghui@ccmu.edu.cn)

Xu Ma, National Human Genetic Resources Center. Life Science Park East road, Changping District, Beijing, China. 102206. [nfpcc_ma@163.com](mailto:nfpcc_ma@163.com)

Table S1 Glycolipids metabolism in the second trimester between two groups.

|  | GDM  (n=47) | Control  (n=47) | P |
| --- | --- | --- | --- |
| Fasting glucose, (mmol/L) | 4.85±0.50 | 4.45±0.31 | 0.034 |
| 1-h glucose, (mmol/L) | 9.83±1.56 | 7.12±1.44 | ＜0.001 |
| 2-h glucose, (mmol/L) | 8.43±1.33 | 6.18±1.10 | ＜0.001 |
| CHO (mmol/L) | 6.38±1.13 | 6.71±1.14 | 0.98 |
| TG(mmol/L) | 3.00(1.49) | 3.01(1.40) | 0.48 |
| HDL(mmol/L) | 1.76±0.31 | 1.87±0.44 | 0.36 |
| LDL(mmol/L) | 3.35±0.97 | 3.56±0.99 | 0.91 |
